# Probability Prediction of Curing Process-Induced Deformation for V-Shape Composite Structures Based on FEM Method and Data Mining

**DOI:** 10.3390/ma17143545

**Published:** 2024-07-18

**Authors:** Guangshuo Feng, Bo Liu

**Affiliations:** 1 School of Mechanical Engineering, University of Science and Technology Beijing, Beijing 100083, China; liubo1@ustb.edu.cn; 2 Beijing Key Laboratory of Lightweight Metal Forming, Beijing 100083, China

**Keywords:** continuous fiber-reinforced composites, curing process-induced deformation, finite element analysis (FEA), data-mining

## Abstract

Continuous fiber-reinforced composites are increasingly used in industry for their superior specific modulus and strength. The curing process-induced deformation (PID) has been a critical problem during manufacturing, which always exhibits dispersed values even if the curing process curve and structural parameters remain consistent. This work conducted probability prediction of PID for V-shape composite structures based on the FEM method and data mining. A sequential coupling thermal–chemical–mechanical coupling FE model is established in ABAQUS. The prediction accuracy of the included angle between two sides is verified by the experimental results. Material parameter uncertainties are considered for V-shape structures with different radii and thicknesses. Based on the dataset from the FE model, a decision tree is established and trained to analyze the sensitivity and to predict the probability distribution of PID. The results show that PID increases with the coefficients of thermal expansion in the in-plane perpendicular fiber direction and out-of-plane normal direction. The data-mining method is accurate enough for the PID prediction, and its efficiency provides an additional calculation option in engineering applications.

## 1. Introduction

Carbon fiber-reinforced thermosetting polymer composites (CFRPs) have been widely used in aerospace, automotive, and marine industries due to their excellent specific stiffness and modulus properties [1,2,3]. The curing process-induced deformation (PID) has been a significant challenge for these structures as dimensional deviations introduced assembly gaps and led to stress concentration at joints [4,5,6]. The PID is normally caused by curing reaction, anisotropic thermal expansion coefficients, and chemical shrinkage. It is more serious for composite structures with complex geometry.

Many researchers have studied the deformation mechanism and value of PID by experimental and numerical methods, in which the numerical method exhibited its advantage in dealing with such highly nonlinear problems [7,8,9,10,11,12,13,14,15]. The curing process numerical model requires a thermal–chemical–mechanical multi-physics analysis, including two critical aspects: thermal–chemical analysis and thermal–mechanical analysis. In the thermal–chemical analysis, the transient heat transfer and temperature field within composite structures are calculated by the curing kinetics model [7,8,9,10]. Most curing kinetic models are established in empirical form. For example, Lee et al. [10] fitted the curing rate as a function of temperature and degree of cure (DoC) using experimental data from a differential scanning calorimeter (DSC) and analyzed the curing behavior of rein. Xiao et al. [11] analyzed the rheological properties of the polymer system to determine a reasonable printing temperature; the nonlinear integral method was applied to establish the model-free kinetics (MFK) of the resin system; reasonable curing process parameters were determined by analyzing the predicted curing degree curves. Based on the thermal–chemical analysis, temperature-dependent mechanical parameters of composite structures can be calculated [12,13,14]. The cure-hardening instantaneous linear elasticity (CHILE) model proposed by Bogetti et al. [15] was one of the most commonly used equations to incorporate temperature and modulus by a semi-empirical function. Al-Dhaheri et al. [8,16] and Luo et al. [17] used the model to describe the strain–stress relationship in the curing process, and the simulation results showed good prediction accuracy compared to experimental results.

As the total coupling thermal–chemical–mechanical model is difficult to realize in finite element simulation, a sequential coupling method is developed by the researchers [9,18,19]. The temperature field is obtained in thermal–chemical coupling model, and then the temperature-dependent mechanical response is calculated. Although the sequential coupling method improves efficiency, it is still time-consuming due to variables updating. Many researchers attempted to predict the mechanical behavior of composite structures during the curing process based on the machine learning method. Stamopoulos et al. [20] designed unidirectional composite panels under different autoclave pressures and established the relationship between autoclave pressure and composite porosity as well as mechanical properties using artificial neural networks. Luo et al. [21] conducted thermo-mechanical coupling finite element analysis on the curing process of asymmetrically laid carbon/epoxy composite materials. Based on the obtained dataset, an artificial neural network for the rapid prediction of curing-induced deformations in different layups of composite materials was established. Aleksendrić et al. [22] integrated genetic algorithms for the optimization of curing regimes, contributing to the formulation of well-suited curing protocols in practical production. Hui et al. [23] used machine learning to replace the general cure kinetics model, finding the relationships between cure kinetics parameters and improving the prediction accuracy of curing behavior.

The objective of this study is to establish an accurate and efficient method for the probability prediction of curing process-induced deformation for V-shape thick composite structures based on sequential coupling multi-field FE method and data mining. Firstly, a sequential coupling of thermo-chemo-mechanical behaviors and the deformation analysis of composites were conducted to acquire the dataset, in which uncertainty material parameters were considered. Then, the parameter sensitivity of PID was analyzed, and probability prediction of curing process-induced deformation for V-shape thick composite structures was achieved. In the end, the prediction accuracy ML-based model was validated, and the distribution of PID for V-shape composite structures was analyzed.

## 2. Governing Equations for Curing Process

The curing process can be divided into three stages based on changes in modulus. In the first stage, the resin is in a liquid or gel-like state with low viscosity; although the chemical reaction and curing process has begun, the material is not yet significantly rigid, and composite materials exhibit a small degree of curing and modulus. In the second stage, the resin transitions from a flexible or viscous state to a more rigid state. Concurrently, curing shrinkage deformation occurs, indicating that the material undergoes volumetric contraction. The composite continues to exhibit an increasing degree of curing and modulus. In the final stage, the resin reaches a high degree of cure, and its mechanical properties stabilize. The modulus remains constant, and no further cure shrinkage occurs, suggesting the resin has achieved its final form and the curing process is nearly complete. This study focuses on the changes in modulus during the second and third stages.

### 2.1. Heat Transfer

The temperature field in composite structures is described by the Transient Fourier anisotropic heat conduction process as follows:

(1) $$\rho_{c}C_{c}\dot{T}=\nabla \left(k\nabla T\right)+\dot{Q}$$

in which $\rho_{c}$, $C_{c}$, and $\dot{T}$ represent the the density of the composite materials, the specific heat capacity of composite materials, and the heating rate in the curing process, respectively. k and $\dot{Q}$ represent the anisotropic thermal conductivity of composite materials and curing heat generation rate, respectively.

### 2.2. Cure Kinetics

The heat generation rate of composite material is affected by the rein-curing reaction rate and can be described by the phenomenological kinetic model as follows:

(2) $$\dot{Q}=\dot{\alpha }\left(1-c_{f}\right)\rho_{r}H_{R}$$

in which $\dot{\alpha }$ is the curing rate; $H_{R}$ is the total exotherm during the resin curing reaction; $c_{f}$ and $\rho_{r}$ are the fiber volume fraction and rein density, respectively.

As the reaction kinetics exhibit different behavior at different stages of the curing process, the reaction rate throughout the entire process cannot be described by a single and simple kinetic model. According to the experimental observation in the previous research [24], the curing rate changed with the degree of curing; the kinetic equation of bismaleimide resin thermosetting rein can be written by a stage function as

(3) $$\dot{\alpha }=\left\{\begin{array}{ll} \left(J_{1}+J_{2}\alpha )(1-\alpha )(0.47-\alpha \right) & \alpha \leq 0.3\\ J_{3}(1-\alpha ) & \alpha >0.3 \end{array}\right.$$

in which $J_{i}=A_{i}exp(-\Delta E_{i}/RT)$. α is the degree of curing for composite; $A_{i}$, $\Delta E_{i}$, and R are the pre-exponential factor, the activation energies of the autocatalytic model, and the universal gas constant, respectively.

### 2.3. Constitute Model

When numerically analyzing the curing process-induced deformation of composite structures, it is critical to describe the Jacobian matrix as it represents the ratio of stress increment to strain increment during each step. In this study, CHILE(α) proposed by Bogetti is used to describe the relationship between rein modulus and degree of curing as [15]:

(4) $$E_{m}=\left\{\begin{array}{c} \frac{E_{e}^{m}}{1000}\;\;\;\;\;\;\;\;\alpha <\alpha_{c1}\\ \frac{(1-\alpha_{mod})E_{e}^{m}}{1000}+\alpha_{modE_{e}^{m}}\;\;\alpha \geq \alpha_{c1} \end{array}\right.,\;G_{m}=\frac{E^{m}}{2(1+\upsilon^{m})}$$

in which $E_{m}$ and $G_{m}$ are the rein elastic modulus and rein shear modulus, respectively; $E_{e}^{m}$ is the fully cured elastic modulus of rein; $\alpha_{c1}$ is the degree of curing at the glass transition point and is set as 0.57 in this study; $\alpha_{mod}$ was assumed to be the DoC value.

After obtaining the modulus of rein, the mechanical constants for composite materials can be calculated by the rule of mixture, as follows:

$$E_{1}^{c}=E_{1}^{f}c_{f}+E^{m}\left(1-c_{f}\right),E_{2}^{c}=\frac{E_{2}^{f}E^{m}}{\left(1-c_{f}\right)E_{2}^{f}+c_{f}E^{m}},G_{12}^{c}=\frac{G^{m}G_{12}^{f}}{G^{m}c_{f}+G_{12}^{f}\left(1-c_{f}\right)},\upsilon_{12}^{c}=c_{f}\upsilon_{12}^{f}+(1-c_{f})\upsilon^{m}$$

Various approaches have been proposed to predict the strain–stress relationships of composite structures. In this study, a semi-empirical model has been widely used due to its rapid computational speed, robust convergence, low computational demand, and high simulation accuracy when compared to physical-based and phenomenological–numerical models. The thermo-mechanical constitute model for composites in the local coordinate system can be written as an increment formula:

(5) $$∆\sigma =\left[C\right]\left(∆\varepsilon -{∆\varepsilon }^{E}\right)$$

in which σ and ε are the stress and strain matrix, respectively; C is the stiffness matrix; $\varepsilon^{E}$ is the expansional strain.

The expansional strain is decomposed into thermal component and chemical component as

(6) $${∆\varepsilon }^{E}={∆\varepsilon }^{th}+{∆\varepsilon }^{sh}$$

in which $\varepsilon^{th}$ and $\varepsilon^{sh}$ are the thermal and chemical shrinkage strains, respectively.

Assuming the composite lamina is unidirectional and transversely isotropic, the Equations (1) and (2) can be rewritten as

$$\left[\begin{array}{c} \begin{array}{c} ∆\sigma_{11}\\ ∆\sigma_{22}\\ ∆\sigma_{33} \end{array}\\ \begin{array}{c} ∆\sigma_{23}\\ ∆\sigma_{31}\\ ∆\sigma_{12} \end{array} \end{array}\right]=\left[\begin{array}{cc} \begin{array}{ccc} C_{11} & C_{12} & C_{13}\\ C_{21} & C_{22} & C_{23}\\ C_{31} & C_{32} & C_{33} \end{array} & \\ & \begin{array}{ccc} G_{23} & & \\ & G_{31} & \\ & & G_{12} \end{array} \end{array}\right]\left[\begin{array}{c} \begin{array}{c} ∆\varepsilon_{11}-∆\varepsilon_{11}^{th}-∆\varepsilon_{11}^{sh}\\ ∆\varepsilon_{22}-∆\varepsilon_{22}^{th}-∆\varepsilon_{22}^{sh}\\ ∆\varepsilon_{33}-∆\varepsilon_{33}^{th}-∆\varepsilon_{33}^{sh} \end{array}\\ \begin{array}{c} ∆\varepsilon_{23}\\ ∆\varepsilon_{31}\\ ∆\varepsilon_{12} \end{array} \end{array}\right]$$

in which the terms in the stiffness matrix are calculated by the anisotropic modulus, as follows:

$$C_{11}=\frac{1-\nu_{23}\nu_{32}}{A}E_{1},\;C_{12}=C_{21}{=C}_{13}=C_{31}=\frac{\nu_{31}+\nu_{21}\nu_{23}}{A}E_{1},\;C_{22}=\frac{1-\nu_{13}\nu_{31}}{A}E_{2},\;\;C_{23}=C_{32}=\frac{\nu_{32}+\nu_{12}\nu_{31}}{A}E_{2},\;C_{66}=G_{12},\;A=1-\nu_{12}\nu_{21}-\nu_{23}\nu_{32}-\nu_{13}\nu_{31}-2\nu_{21}\nu_{32}\nu_{13}.$$

The thermal strain can be calculated by the coefficients of thermal expansion and temperature increment, as follows:

(7) $$\Delta \varepsilon^{th}=\left[\begin{array}{c} ∆\varepsilon_{11}^{th}\\ ∆\varepsilon_{22}^{th}\\ ∆\varepsilon_{33}^{th} \end{array}\right]=\left[\begin{array}{c} a_{1}\\ a_{2}\\ a_{3} \end{array}\right]\Delta T$$

in which $\alpha_{11}$, $\alpha_{22}$, and $\alpha_{33}$ are the coefficients of thermal expansion for different directions.

Curing shrinkage can be simplified as a temperature load aroused by the curing reaction of thermoset resin in the finite element simulation, as follows:

(8) $$\Delta \varepsilon^{sh}=\left[\begin{array}{c} ∆\varepsilon_{11}^{sh}\\ ∆\varepsilon_{22}^{sh}\\ ∆\varepsilon_{33}^{sh} \end{array}\right]=\left[\begin{array}{c} b_{1}\\ b_{2}\\ b_{3} \end{array}\right]\Delta T^{sh}$$

in which $\Delta T^{sh}$ is the equivalent temperature difference.

## 3. Data Mining Algorithms

It is unrealistic to obtain the dataset for data mining through numerous experiments. Data mining that can discover patterns and knowledge within a dataset by statistics, machine learning, and data analysis methods is used in this study. Data mining models progressively reveal latent structures and relationships in the data, uncovering patterns and trends relevant to the objectives, thereby facilitating predictive analytics. Decision trees are a commonly employed data mining method for agent modeling and decision-making. They utilize a tree-like structure to represent a series of decision rules for classifying or regressing input data. Key features of decision trees include their tree-like structure, nodes, and branches. Each node represents a specific test or decision rule for the input data, while each branch indicates different outcomes under the test conditions. The construction process of a decision tree involves selecting optimal features and corresponding split points to achieve the best data classification or regression performance at each node.

### 3.1. Decision Tree Algorithms

**Figure 1:**
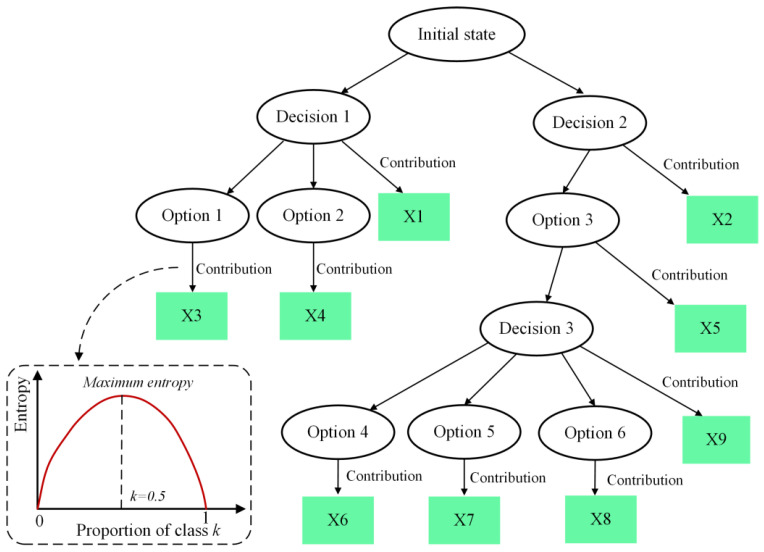
Diagram of decision tree.

In this study, based on the hierarchical structure of neural network characteristics of decision trees, we built decision trees to realize sensitivity analysis and probability prediction. Figure 1 shows the schematic diagram of a decision tree. Nodes serve to represent the various states or options. In the construction of the decision tree, decision-making processes are realized through the assessment of optimal attributes associated with options. The quantification of the purity of sample categories within the node options is measured by information entropy. Assuming that the proportion of class *k* samples in dataset *D* is $p_{k}(k=1,2,\cdots ,\left|y\right|)$, the information entropy of D is defined as

(9) $$Ent(D)=-\sum_{k=1}^{y}p_{k}\log_{2}p_{k}$$

When the proportion $p_{k}$ approaches 0 or 1, the information entropy tends to 0 because the sample set tends to be pure. When $p_{k}$ is close to 0.5, the information entropy reaches the maximum, indicating the highest uncertainty of the data. The decision tree is employed for computing the contribution of input parameters.

To assess the input parameter contributions, a dataset comprising nine input parameters and corresponding decision outcomes was collected. This dataset was used to train a decision tree model, partitioned into training and testing sets. The decision tree was constructed using the training set, ensuring that the model considered the contribution of each input parameter during the tree-building process. Each of the nine input parameters was introduced at various nodes of the decision tree, particularly decision and leaf nodes, where they could influence decision outcomes differently. An analysis was conducted to determine the impact of each input parameter across different nodes of the decision tree. The objective was to ascertain the direction (positive or negative) and the relative magnitude of each parameter’s contribution to the decision-making process.

### 3.2. Implementation of Decision Tree

**Figure 2:**
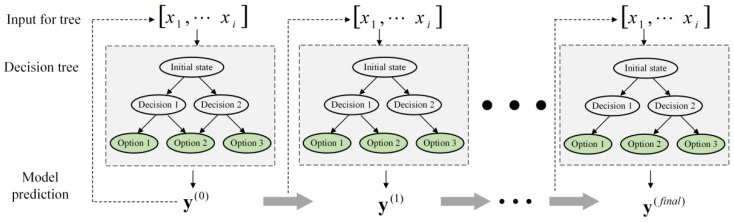
Diagram of decision tree trains and predicts.

The decision tree algorithm was implemented by Python and Scikit-learn libraries. Figure 2 shows how the model trains and predicts. Firstly, the input dataset was normalized and standardized for converting the input data. Secondly, the input dataset was divided into two parts, a training set and a test set, each accounting for 70% and 30%. Then, the training process of the decision tree was conducted until the R2 value between the predicted value and simulation value was less than the threshold value.

## 4. Results and Discussion

### 4.1. Finite Element Modeling

#### 4.1.1. Implement of Multi-Field Finite Element Model

**Figure 3:**
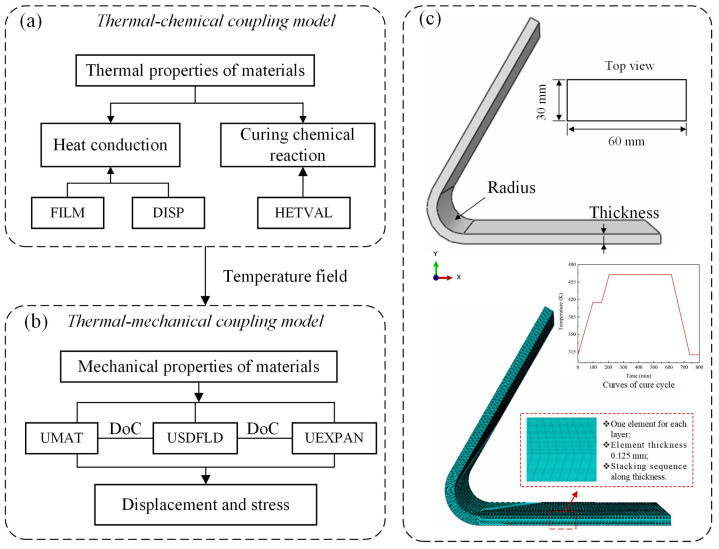
(**a**) Flow chart of thermal–chemical coupling model; (**b**) flow chart of thermal–mechanical model; (**c**) geometry model of V-shape composite structure.

The sequential thermal–chemical–mechanical coupling model was implemented using ABAQUS FE software 2017. This model consists of three main modules: geometry, chemical heat transfer, and stress analysis. In the geometry module, a V-shape composite structure with a 60° corner angle and 8 mm corner radius was defined (Figure 3a). The model was discretized into 24 layers, each with a thickness of 0.125 mm, resulting in a total of 103,680 elements. The chemical heat transfer module simulated temperature and degree of cure (DoC) distributions. The temperature field obtained for each increment served as input for the stress analysis module, where strain/displacement distributions of the composite structure were calculated. Within the chemical heat transfer module, the element type used was an eight-node linear heat transfer brick (DC3D8), with temperature boundary conditions applied. Subsequently, in the stress analysis module, an eight-node linear brick element (C3D8) was utilized to prevent shear self-locking phenomena.

#### 4.1.2. FE Model Validation

**Figure 4:**
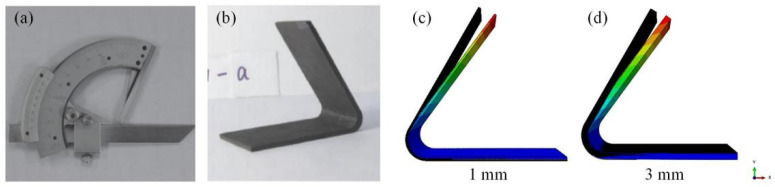
(**a**) Protractor [25]; (**b**) V-shape specimen [25]; (**c**) simulation results of V-shape specimen with 1 mm thickness; (**d**) simulation results of V-shape specimen with 3 mm thickness.

The V-shaped structure with T700 carbon fiber and QY9611 bismaleimide resin was cured in an autoclave. After the curing process was completed, the angular rebound deformation of the composite V-shape specimens was measured using a protractor with a graduation value of 2 min (Figure 4a,b) [25]. In the finite element model, the deflection angle was calculated using the tangent of the deformation and the side length. The curing-induced deformation from experimental and simulation results are compared in Table 1. The maximum error between simulation and experimental results was 7%, which indicated that the model was accurate enough to build datasets for training the decision tree model.

### 4.2. Probability Prediction of Curing Process-Induced Deformation

#### 4.2.1. Uncertain Material Parameter Qualifications

With the established FE model, the deformation of composite structures can be calculated under the given material parameters in Equations (1)–(8). These parameters were divided into three categories: thermal expansion coefficients, chemical shrinkage coefficients, and mechanical performance parameters. Normally, they were obtained from specimen experiments or set based on the engineers’ experience [25,26]. Uncertain factors such as material suppliers, testing resources, or testing errors are inevitable to cause the parameter value fluctuation. In order to quantify this phenomenon, statistical methods were used, and probability distribution functions (PDF) were established. For composite structures, the PDF of mechanical parameters followed a Gaussian distribution [24]. Assumed thermal expansion coefficients and chemical shrinkage coefficients also followed the Gaussian distribution; material parameters used for the FE model in this study were listed in Table 2 [25].

#### 4.2.2. Sensitivity Analysis

The structural geometry parameters are easy to measure; thus, the uncertainty of material parameters is considered in this study. Materials parameters were extracted in the design space in Table 2 and written into input files by Python 3.0. Detailed geometry parameter conditions were listed in Table 3: the thickness of the composite was 1 mm, 2 mm, 3 mm, and 4 mm; the radius of the V-shape composite was 6 mm, 8 mm, 10 mm, and 12 mm. A total of 1400.inp files were generated for submitting ABAQUS 2017 calculations.

**Figure 5:**
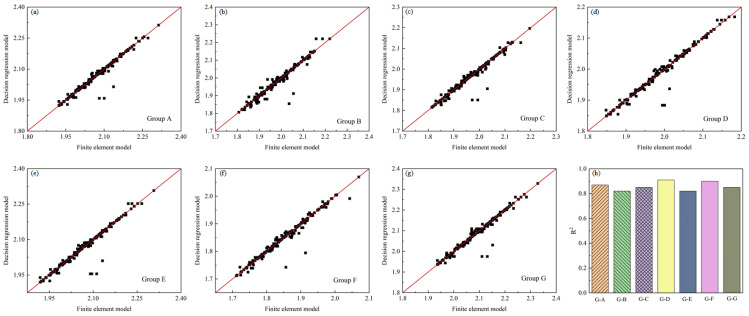
Comparison of finite element method and surrogate models.

After obtaining the input files, the maximum displacement of the V-shape composite structures was extracted at the last increment step and written into the dataset to train the decision tree. Seven distinct regression models were trained for sensitivity analysis and probability prediction. Figure 5 depicts a scatter plot of finite element analysis results and regression model predictions for the employed test dataset conditions, with the red line representing a perfect fit. The proximity of scattered points to the perfect fit line indicates the accuracy of the surrogate models. Figure 5h illustrates the R-squared values for all surrogate models trained on deformations induced by curing. All R-squared values exceeding 0.8 suggested an acceptable accuracy for surrogate models.

RSME (Root Mean Square Error) and MSE (Mean Squared Error) were also used to evaluate the accuracy of the surrogate model. A smaller RMSE and MSE indicated a higher accuracy in the model predictions. The regression model in this study showed a good prediction capacity (Table 4).

**Figure 6:**
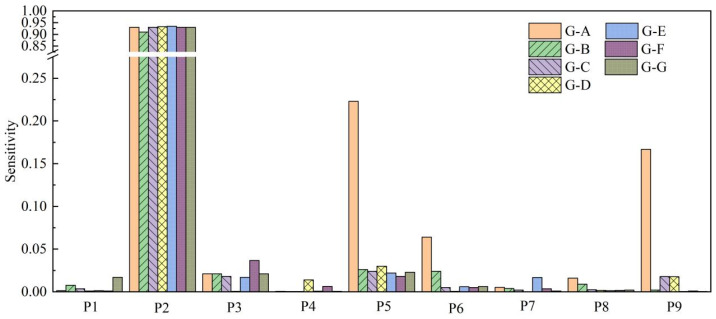
Sensitivity analysis results for different groups.

The sensitivity analysis results are shown in Figure 6. The PID of the V-shape structure was most sensitive to the coefficients of thermal expansion in the in-plane perpendicular fiber direction and out-of-plane normal direction (P2 in Table 2) for different structures. This sensitivity arises primarily due to two reasons. Firstly, the temperature field of composite material components is composed of the external process curve temperature field and the exothermic heat released from the curing reaction. The exothermic heat release during the curing reaction of the composite material is primarily associated with the chemical reaction of the resin. Secondly, the chemical expansion coefficient of the resin is much greater than that of the fibers and is directly related to the deformation of the structure.

**Figure 7:**
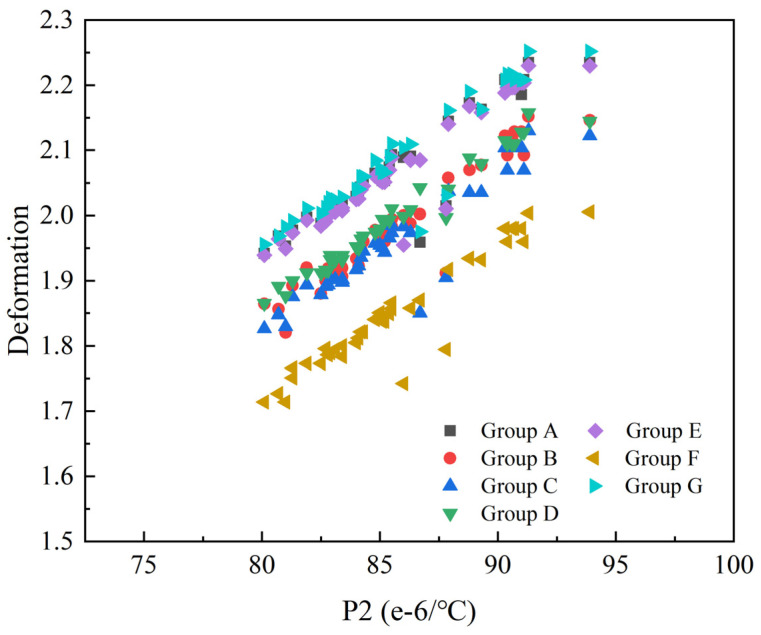
The relationship between P2 and curing-induced deformation.

The relationship between PID and P2 (Table 2) is illustrated in Figure 7. The two variables exhibited a positive correlation, indicating that the PID increased with the increasing thermal expansion coefficients. This phenomenon emphasized that the thermal expansion properties played a critical role in governing the PID of composite structures. Understanding and quantifying these sensitivities are crucial for optimizing the design and predicting the performance of composite structures during the curing process.

#### 4.2.3. Probability Prediction of Curing Process-Induced Deformation

**Figure 8:**
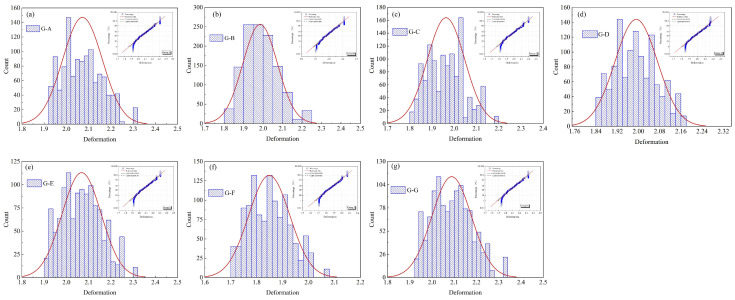
Distribution of curing-induced deformations for V-shape composite structures.

Figure 8 presents the distribution of PID for composite structures with different geometry. The inset graphs at the top right depicted the upper and lower bounds of the deformation magnitudes. The PID showed a Gaussian normal distribution. For Group A, over 99% of curing-induced deformations are distributed between 1.8 mm and 2.4 mm. For Group F, over 99% of curing-induced deformations are distributed between 1.6 mm and 2.1 mm.

**Figure 9:**
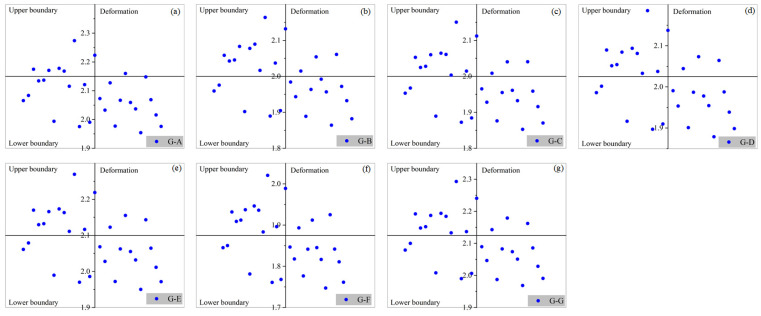
Distribution of PID from data mining.

In order to verify the reliability of PID probabilistic prediction, 30 additional sets of data were calculated by numerical model (Figure 9). These numerical PID values are uniformly distributed within the upper and lower bounds determined through data mining, highlighting the generality and feasibility of the employed methodology.

By employing a combination of multi-field finite element simulation and data mining techniques, the prediction of curing-induced deformations in composite materials has been successfully constrained within a specific dataset. Considering the distribution and upper/lower limits of material mechanical properties, we derived the distribution and threshold values of deformation. During the manufacturing phase, effective quality control measures can be implemented through real-time monitoring and prediction of deformations in the curing process of composite materials. This contributes to the reduction of manufacturing defects, enhancing product consistency and quality. This approach not only ensures the production of high-quality goods but also optimizes manufacturing processes, reducing resource waste and lowering production costs.

Accurate prediction of the range of curing-induced deformations in composite materials is crucial for driving the development of new materials. By gaining in-depth insights into the deformation behavior of composite materials during the curing process, engineers can design more advanced and reliable materials based on this understanding. This not only improves material performance but also expands the application areas of materials, fostering technological innovation.

In summary, the engineering significance of accurately predicting curing-induced deformations in composite materials lies in optimizing engineering design, improving product quality, and reducing manufacturing costs. This predictive approach not only aids in the current improvement of engineering practices but also provides a foundation for future innovations. Through the integration of advanced simulation techniques and data analysis methods, engineers can address challenges related to curing-induced deformations in composite materials more reliably and efficiently, contributing to the sustainable development of the engineering field.

## 5. Conclusions

This work proposed a prediction method to predict the probability of PID for V-shape composite structures based on the FEM method and data mining. A sequential coupling thermal–chemical–mechanical coupling FE model was established by the user defined subroutine and verified the experimental results. A decision tree was built and trained based on datasets from the FE model. The sensitivity and probability distribution of PID were analyzed, as aforementioned. The results showed that the accuracy of the sequence coupling FE model is enough to simulate the PID of V-shape composite structures. The maximum simulation error is less than 5% for a V-shape specimen with 3 mm thickness. The curing-induced PID is most sensitive to the coefficients of thermal expansion in the in-plane perpendicular fiber direction and out-of-plane normal direction, regardless of the thickness and radius of the V-shape structure. The data-mining method is accurate enough for the PID probability prediction, and its efficiency provides an additional calculation option in engineering applications. The PID of the V-shape specimen showed Gaussian normal distribution. In a future study, the effect of curing parameters on mechanical properties will be further discussed.

## Figures and Tables

**Table 1:** Experimental and numerical results comparison for V-shaped composite parts.

Part ID	Thickness	Radius	${∆\theta }_{exp}$	${∆\theta }_{sim}$	Error
Sample (a)	1	8	2.15	2.00	7%
Sample (b)	3	8	1.81	1.89	4.4%

**Table 2:** Statistical properties of input uncertain parameters.

	Sq.	Parameters	Unit	Lower Limit	Upper Limit	Mean Value	SD
Thermal expansion	P1	a_1_	/°C	0.7 × 10^−6^	1.3 × 10^−6^	1.0 × 10^−6^	0.1 × 10^−6^
	P2	a_2_ = a_3_	/°C	79.1 × 10^−6^	94.9 × 10^−6^	85.3 × 10^−6^	3.5 × 10^−6^
Chemical shrinkage	P3	b_1_	/	0.7 × 10^−4^	2.8 × 10^−4^	1.67 × 10^−4^	0.4 × 10^−4^
	P4	b_2_ = b_3_	/	6.2 × 10^−4^	11.7 × 10^−4^	8.81 × 10^−4^	1 × 10^−4^
Mechanical parameters (Glassy)	P5	E_1_	GPa	119.9	139.7	130	8.5
	P6	E_2_ = E_3_	MPa	155.9	173.3	165	10.2
	P7	G_12_ = G_13_	MPa	33.1	44	40	1.8
	P8	G_23_	MPa	31.1	37.7	35	1.5
	P9	V_12_	/	0.12	0.38	0.25	0.005

**Table 3:** Parameters for different groups.

	Group A	Group B	Group C	Group D	Group E	Group F	Group G
Thickness	3 mm	1 mm	2 mm	4 mm	3 mm	3 mm	3 mm
Radius	6 mm	6 mm	6 mm	6 mm	6 mm	10 mm	12 mm

**Table 4:** RSME and MSE for surrogate model.

	Group A	Group B	Group C	Group D	Group E	Group F	Group G
RSME	0.0277	0.0339	0.0291	0.0224	0.0333	0.0225	0.0301
MSE	0.0007	0.0011	0.0008	0.0005	0.0011	0.0005	0.0009

## Data Availability

The original contributions presented in the study are included in the article, further inquiries can be directed to the corresponding author.
